# Revealing users’ experience and social interaction outcomes following a web-based smoking prevention intervention for adolescents: A qualitative study

**DOI:** 10.1371/journal.pone.0223836

**Published:** 2019-10-17

**Authors:** Georges Elias Khalil, Hua Wang, Karen Sue Calabro, Alexander V. Prokhorov

**Affiliations:** 1 Department of Behavioral Science, the University of Texas MD Anderson Cancer Center, Houston, Texas, United States of America; 2 Department of Communication, University at Buffalo, the State University of New York, Buffalo, New York, United States of America; The MetroHealth System and Case Western Reserve University, UNITED STATES

## Abstract

**Background:**

Tobacco smoking remains a public health problem among adolescents in the United States. While Web-based interventions for smoking prevention have been successful at the individual level, there is still an urgent need to understand their engagement capabilities and their effects at the social level. In the current study, we aimed to (1) learn about adolescents’ subjective experience with a Web-based program called a smoking prevention interactive experience (ASPIRE), (2) obtain suggestions for improvement in ASPIRE content, (3) identify psychological outcomes of ASPIRE, and (4) explore outcomes of social interaction.

**Materials and methods:**

After a randomized controlled trial with 110 adolescents, 20 adolescent users of ASPIRE, aged 11–18, were randomly selected to participate in one-on-one interviews at four after-school programs in Houston, Texas. Interviews involved questions concerning adolescents’ experience with the intervention. Qualitative data were coded and analyzed using a constant comparison approach for the generation of themes.

**Results:**

Describing their experience with ASPIRE, participants expressed comfort in material that is tailored to their demographic and preferred interactive activities over entertaining videos. Presenting suggestions for improvement, participants mainly reported the need to include gaming features into ASPIRE. Presenting psychological outcomes, they expressed emotional engagement in the program, shifts in attitudes and beliefs, and unwillingness to smoke. Finally, as outcomes of social interaction, participants reported engagement with others in discussions about tobacco and their need to hold smokers accountable for their actions.

**Conclusions:**

Adolescents’ reports moved from their individual experience with ASPIRE to their active interactions with family members and friends and their attempt to persuade others to quit smoking. Future Web-based programs for adolescents may be designed with tailoring and game play in mind, in order to provide mobilization skills and foster social interactions against smoking.

## Introduction

### Background

Despite the vast evidence on the negative consequences of tobacco use, smoking remains a public health concern among adolescents [1–4]. Risky behaviors such as tobacco smoking are learned during this age period [2], and adolescence is a time when smoking behavior increases [1, 5, 6]. It is hence crucial to communicate to adolescents the risks of smoking. Capturing the attention of adolescents can be challenging, especially if the objective is to prevent the use of tobacco products.

Previous meta-analyses and systematic reviews of all types of available tobacco prevention and cessation programs for youths and adolescents have been thoroughly conducted to examine best practices in tobacco prevention and treatment. Results of such reviews have indicated that tailored content that includes social influence and social competence components is most successful in driving both prevention and treatment of tobacco use [7, 8]. Compared to other currently available strategies for smoking prevention, one particularly innovative strategy has been the implementation of Web-based interventions [9, 10]. Several of these interventions have shown success among adolescents [7–10]. One example is a program called A Smoking Prevention Interactive Experience (ASPIRE). A randomized controlled trial conducted in 2002 has shown that ASPIRE can prevent smoking initiation by 18-month follow up [11]. Adolescents who received the ASPIRE intervention were less likely to initiate smoking compared with adolescents who received a booklet about the effects of smoking.

### Gaps in knowledge

Currently, there is little knowledge concerning the best ways to improve the features of Web-based interventions and understand how they can drive outcomes beyond smoking prevention at the individual level. First, available web-based interventions for smoking prevention deserve improvement by designing more modern versions that make use of today’s technology features. Such interventions tend to be technologically rudimental [11, 12] and restricted by the challenging environment of the school setting [13]. For instance, ASPIRE was designed in 2002 and featured aspects popular for that period (e.g., dated fashion styles, furnishings, and early technological devices). The program was also marked by limited technology features (e.g., interacting with activities and watching videos of smokers’ testimonies). Since then, a cultural evolution has transpired, marked by new technology features for Web-based health interventions, including social media, game play, and tailoring capabilities. As a result, there is a need to investigate ways to advance the current version of ASPIRE in order to boost its success.

Second, while programs have been designed to drive behavior change at the individual level, their ability to mobilize adolescents and motivate them to engage in social discussions against smoking is still not known. Although this phenomenon has been observed for other health outcomes [14–16], it is still open for investigation with smoking prevention programs [12]. For instance, ASPIRE was designed to allow human-computer interaction using interactive activities that reveal the negative consequences of smoking. However, the activities in ASPIRE do not allow adolescents to interact with each other. Nevertheless, previous research has shown that entertainment-based interventions can be designed to stimulate interpersonal communication supportive of healthy behaviors. For instance, in Africa and Asia, entertainment-based interventions for family planning allowed individuals to develop mobilization skills and promoted conversations with loved ones about contraceptive use [14, 15]. Also, entertainment-based interventions for the promotion of organ donation in the United States have encouraged positive discussions about organ donation [16]. As a result, Web-based programs for tobacco prevention may be capable of promoting social interaction among adolescents through their entertaining and interactive components.

### Theoretical framework

**Fig 1:**
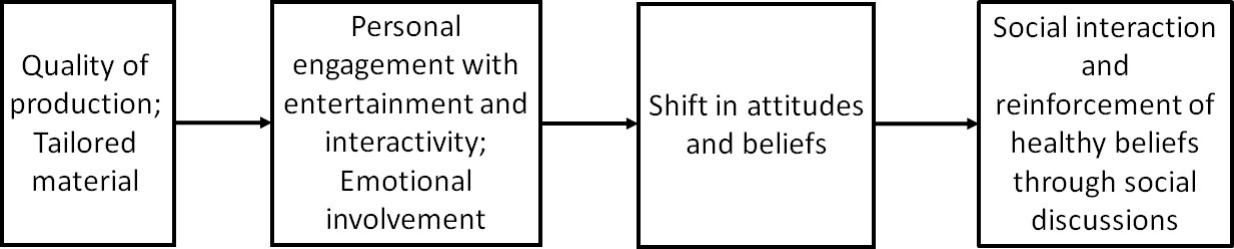
The extended-elaboration likelihood model. This figure presents an adapted depiction of the extended-elaboration likelihood model (E-ELM) with a shift from personal to social outcomes.

The potential ability of Web-based interventions to promote social interaction is supported by the extended elaboration likelihood model (E-ELM). This model posits that individuals’ experience with entertainment-based interventions can influence attitudes, enhance persuasive effects, and stimulate social reinforcement through interpersonal discussions supportive of healthy behaviors (Fig 1) [17, 18]. Similarly, the capacity of entertainment-based interventions to prompt individuals to engage in health-related discussions may be valuable in the context of smoking prevention. While ASPIRE was not purposely designed for the promotion of positive discussions, it is still not known if adolescents’ experience in ASPIRE may promote social outcomes. Adolescents who engage in discussions against smoking may reaffirm their decision not to smoke and in turn become opinion leaders, diffusing messages against tobacco smoking to their social network of family members and friends. As a result of such potential phenomenon, the current study will qualitatively investigate ASPIRE’s ability to promote social interaction outcomes.

### Previous qualitative investigations

Previous work qualitative studies have been pertinent to both smoking prevention and cessation, attempting to answer a variety of research questions intended to young populations. First, a vast number of qualitative studies have examined the challenges that young populations face when considering smoking cessation programs [19]. Their typical reaction to smoking cessation or prevention tends to involve their report of a lack of time and energy to participate in treatment or membership to programs that may be outside their curriculum [19]. This suggests that smoking cessation and prevention programs may be best implemented in a school-setting through established curricula. Second, qualitative studies among adolescents have highlighted patterns in smoking outcomes [20, 21]. Third, previous qualitative examination of Web-based smoking prevention interventions has yielded valuable insights with respect to design recommendations [22]. In particular, the results of such research indicated that the use of websites is attractive to adolescents and the application of interactive tools online is favorable. Nonetheless, qualitative work is still limited in the study of adolescents’ experience with web-based programs for smoking prevention or cessation, and the identification of potentially novel smoking-related psychosocial and behavioral outcomes, such as social interaction.

### Study aims

A part of the mission of the National Institute on Drug Abuse (NIDA) is to acquire new knowledge to develop prevention interventions that target the initiation of drug use [23]. In response, the current study supports such a mission through a qualitative investigation of adolescents’ experience with ASPIRE. The current study aimed to examine adolescents’ experience based on the E-ELM, and then extend our understanding of the user experience through emerging new themes. Through qualitative interviews, we aimed to (1) learn about adolescents’ subjective experiences with ASPIRE, (2) obtain suggestions for improving ASPIRE content, (3) identify psychological outcomes of ASPIRE, and (4) explore social interaction outcomes.

## Materials and methods

### Study setting and the ASPIRE program

In this descriptive qualitative study, we draw findings from interviews following a randomized controlled trial titled *ASPIRE Reactions* (Registered at ClinicalTrials with the registration number: NCT02469779 [24]). ASPIRE Reactions was conducted in 2015 to evaluate the effect of adolescents’ emotional and cognitive experience with ASPIRE on intention to smoke [25]. The trial randomly selected 4 after-school programs located at financially disadvantaged regions of Houston, Texas, including the Boys and Girls Clubs (2 sites), the Salvation Army Boys and Girls Clubs (1 site), and the Young Men's Christian Association (YMCA; 1 site). At these after-school programs, adolescents engage in educational and extracurricular activities that foster their mental and physical health. Adolescents are provided their own private room and a program staff monitors their activities. Among such activities is the engagement in health promotion programs such as ASPIRE.

**Fig 2:**
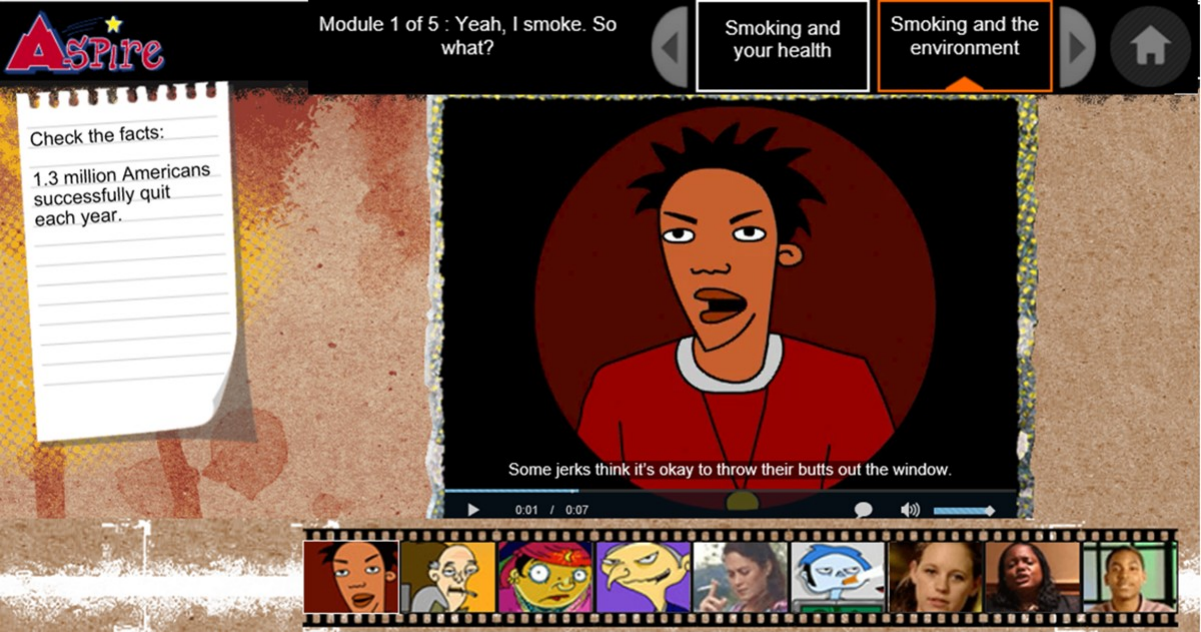
A screenshot of the aspire platform.

ASPIRE includes 4 sessions, each lasting approximately 45 minutes. In design, ASPIRE uses interactivity and entertainment to engage adolescents with text, animations, videos, and activities (Fig 2). Text mainly involves educational facts about tobacco. Through videos and activities, adolescents watch cartoon animations and testimonies from other adolescents and explore health information in virtual environments. In content, ASPIRE development was guided by the transtheoretical model (TTM) of the stages of change. The TTM posits that adolescents move from a stage of pre-contemplation to contemplation, action, and then maintenance of healthy behavior [26]. In ASPIRE, processes of change allow adolescents to move from one stage to another. ASPIRE works to maintain adolescents at a smoke-free stage by presenting information on “celebrating a healthy choice”, “remembering reasons for not smoking”, “smoking and social life”, “smoking and health”, “smoking and the environment”, “steps to quit smoking”, “dealing with slips”, “health from quitters”, “social temptations”, “psychological mood”, “addiction”, and “learning to talk about stress”.

### Inclusion/Exclusion criteria

Adolescents between the ages of 11 and 18 years who attended the after-school program at least twice per week were eligible for the study. Adolescents were not eligible if they were smokers (i.e., have smoked a cigarette, cigar, or hookah in the past year). Also, to be eligible to participate in the interviews, participants must have completed all sessions of ASPIRE during the ASPIRE Reactions trial.

### Recruitment and sampling strategy

**Fig 3:**
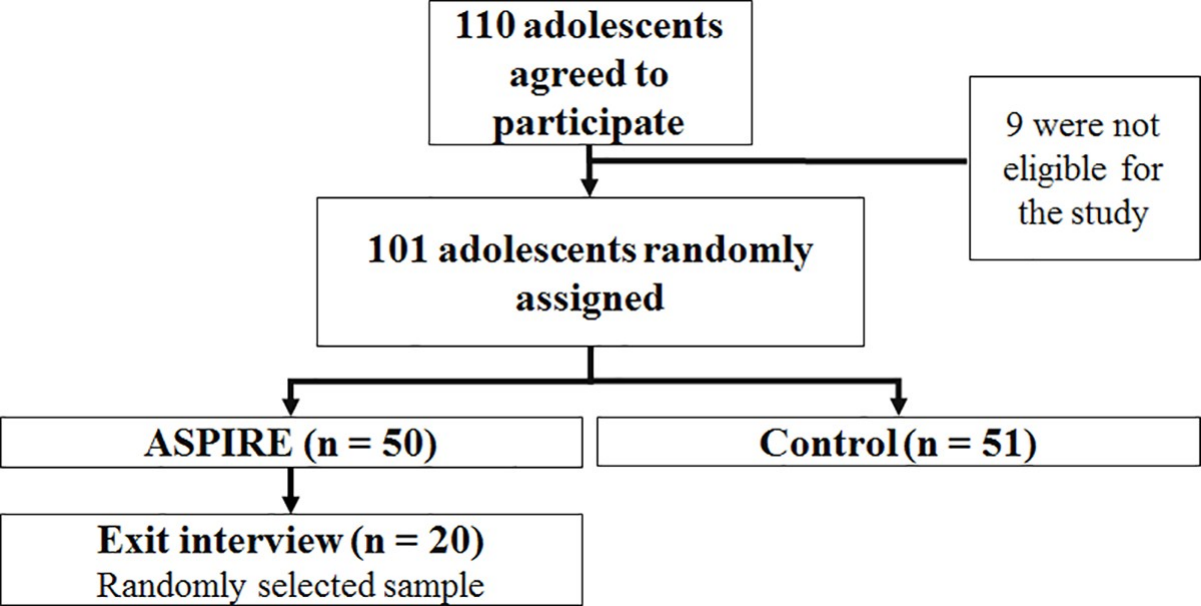
Study flow diagram leading to the exit interview. ASPIRE indicates the main intervention; the control condition involves a text-based version of the main health information related to nicotine and tobacco, presented on the computer screen.

Of 110 interested adolescents, 101 were eligible for the trial. During the trial, adolescents were randomized to receive ASPIRE or a control condition. For the current study, participants from the ASPIRE condition were randomly selected to take part in an exit interview upon completion of the trial (Fig 3). All selected participants were approached face-to-face and agreed to take part in the interview. Recruitment of interview participants continued until information saturation was reached.

### Ethics statement

The current manuscript adheres to the consolidated criteria for reporting qualitative research (COREQ; S1 File) [27], and the study was reviewed and approved by the Institutional Review Board (IRB) from The University of Texas MD Anderson Cancer Center. The interviews were conducted face-to-face in private rooms at the after-school program where adolescents felt safe and relaxed. Adolescent participants were informed about study purpose and procedure and consented for this study by providing signed written informed assent forms. Parental permission was also obtained for adolescent participation. Participants were required to maintain the confidentiality of their identity and the identity of other participants. They were told that they may withdraw from the interview at any time or ask the interviewer to change the subject if they felt uncomfortable during the interview. Participants were given the chance to take breaks during the interview. For further anonymity and as requested by the after-school programs, quantitative information obtained from the trial was not linked to qualitative information obtained during the interviews. Only gender information was obtained during the interviews. In case adolescents disclosed personal or identifying information, such information was not retained within the transcripts. After an interview, a $15 gift card was provided as compensation for participating.

### Interview instrument

The instrument was initially developed based on the extended elaboration likelihood model (E-ELM), and then it was adapted to identify potentially new concepts. Following a pilot study to test an initial instrument with 10 adolescents, a final set of 11 interview questions was developed. As part of the instrument, adolescents were asked to identify videos and activities that were most or least appealing to them, explain what makes such videos and activities appealing or not appealing, and describe what they would add, remove, or edit in ASPIRE if they were given the opportunity. Finally, participants were asked what they have done or plan to do as a result of the ASPIRE experience. Details of the qualitative data collection tool can be found under the supporting information file [S2 File].

### Data collection

Data collection involved one lead researcher at the rank of a postdoctoral fellow (GEK, gender: male, credentials: M.P.H., Ph.D.) and one trained research assistant (NM, gender: female, credentials: B.Sc.). Both interviewers had full training in the principles of qualitative research. There were no interviewer-related biases identified in this study. Logistics used included one vehicle for transportation to each after-school program site, one audio recorder, the interview instrument, and the initial codebook. Prior to the interviews, a relationship of one month was established with the participants in order to build trust and freedom in information-sharing. This relationship was established through the randomized trial that was conducted prior to the interviews. The interviewers were present during the trial data collection. In addition, the interviewers volunteered as staff members in the after-school program. Such volunteering included engaging in summer camp activities with the adolescents. Rapport building allowed the reduction bias and facilitated the discussion process during the interviews. Participants entered the interview room individually and were seated comfortably in a living room setting. No one else was present during the interview, besides the participant and the interviewer. Participants were briefed on the purpose of the study and understood that it was a research project on their experience with the website ASPIRE. They were asked to refrain from disclosing personal identifying information. GEK conducted semi-structured discussions using the interview questions. In addition to key questions, the discussions centered on ways to improve ASPIRE and outcomes as a result of ASPIRE. Participants were given the freedom to answer questions at their own pace. Interviews took approximately 15 minutes in length. In addition to audio-recording, the research assistant took notes of the main ideas conveyed by participants during the interview. Repeat interviews were not carried out in this study.

### Qualitative analysis

The theoretical underpinnings to be studied involve the examination of potential concepts that can extend E-ELM, by examining how adolescents’ personal experience with ASPIRE transitions toward a social experience. Interview recordings were transcribed by the research team and transcripts were analyzed for emergent themes by two coders. Only one of the two interviewers was also a coder. As a result, at least one coder was familiar with the transcripts. This led to an efficient coding process with at least one researcher who has had firsthand experience with the interviews. The second coder was not involved in the interview process, thereby minimizing potential coding bias. The coders used both a constant comparison approach with open coding, as well as a grounded theory approach [28]. A first version of the codebook was developed based on expectations from the E-ELM. Then, open coding with thematic content analysis was conducted in order to identify new themes. Following grounded theory, as new themes emerged, the codebook continued to evolve, as part of an iterative and inductive process. As each transcript was reviewed, recurrent topics and relevant quotes were identified and organized to form a list of common themes. In other words, an iterative process was conducted, generating codes, and in turn, new themes were identified [28]. This process continued until thematic saturation was reached. Partially supportive of the grounded theory, retrieved themes are deemed appropriate as they provide new concepts that extend the E-ELM. The second supporting information file [S3 File] presents the generated final codebook. Cohen’s Kappa coefficient [29] and Krippendorff’s alpha [30] were measured in order to determine the percentage of inter-coder agreement and the alpha coefficient of agreement between the two coders after the first week of coding. During a one-month coding process, the two coders met weekly, and divergent ratings were identified and discussed in order to reach 100% agreement. During this analysis, negative cases were identified and coded when they contradicted a common theme. Participants were not asked to provide feedback on the findings, and the transcripts were not returned to participants for comments or correction. Microsoft Word and Excel were used to manage the data. Following the coding process, the coders identified common themes that are each defined by a set of codes. Such common themes among participants formed the results of the current study.

## Results

All adolescents who were approached for the study agreed to participate in the interview and completed their interview session. Twenty participants were needed to reach thematic saturation. Participants (65% male) had an age ranging between 11 and 18 year. Participants in the ASPIRE Reactions trial who received ASPIRE also ranged from 11 to 18 years in age, and were 61% male. Table 1 presents a description of the main themes and their categories. Thematic analysis revealed three overarching themes: (1) the user experience with ASPIRE, (2) psychological outcomes of ASPIRE, and (3) social interaction outcomes. The qualitative data can be found under the third supporting information file (S4 File). Table 2 presents themes under each category and inter-coder agreement following the first week of coding. At this first week of coding, agreement based on Cohen’s Kappa ranged between 40.45% and 95.65%, indicating acceptable initial agreement, before reaching 100% by the last week of coding. Agreement based on Krippendroff’s alpha ranged between 0.56 and 0.89 (Table 2).

**Table 1:** Theme descriptions.

Themes and Categories	Descriptions
**User experience with ASPIRE**	
** Tailoring program characters**	Information targeting users’ demographics
** Preference for interactivity over entertainment**	Enthusiasm toward interactive features (clicking behavior, uncovering hidden information, exploring virtual environments)
** Suggestions for future design**	Creative ideas to improve ASPIRE
**Psychological and Health Outcomes of ASPIRE**	
** Emotional outcomes**	Expression of emotions as a result of ASPIRE content
** Shift in attitudes and beliefs**	Expression of change in opinion regarding smoking
** Unwillingness to smoke**	Report of the rejection of smoking
**Social Outcomes of ASPIRE**	
** Social accountability**	Hold smokers accountable for their actions
** Interpersonal discussion and persuasion**	Engagement in discussions with others against smoking and attempt to convince others that they should not smoke

**Table 2:** Inter-coder reliability testing for each theme.

Categories and Themes	Cohen’s Kappa^a^	Krippendroff’s Alpha
User experience with ASPIRE		
Tailoring program characters	82.61%	0.65
Preference for interactivity over entertainment	40.45%	0.78
Suggestions for future design	91.30%	0.82
Psychological and Health Outcomes of ASPIRE		
Expression of emotions as a result of experience	91.30%	0.80
Shift in attitudes and beliefs	82.61%	0.56
Unwillingness to smoke	86.96%	0.71
Social Outcomes of ASPIRE		
Interpersonal discussion and persuasion	91.30%	0.78
Social accountability	95.65%	0.89

Values are calculated after the first week of coding. An agreement of 100% was reached by the final session of coding.

^a^All Cohen’s Kappa values were statistically significant with a p-value lower than 0.001.

### Major theme I: User experience with ASPIRE

#### Tailoring

**Fig 4:**
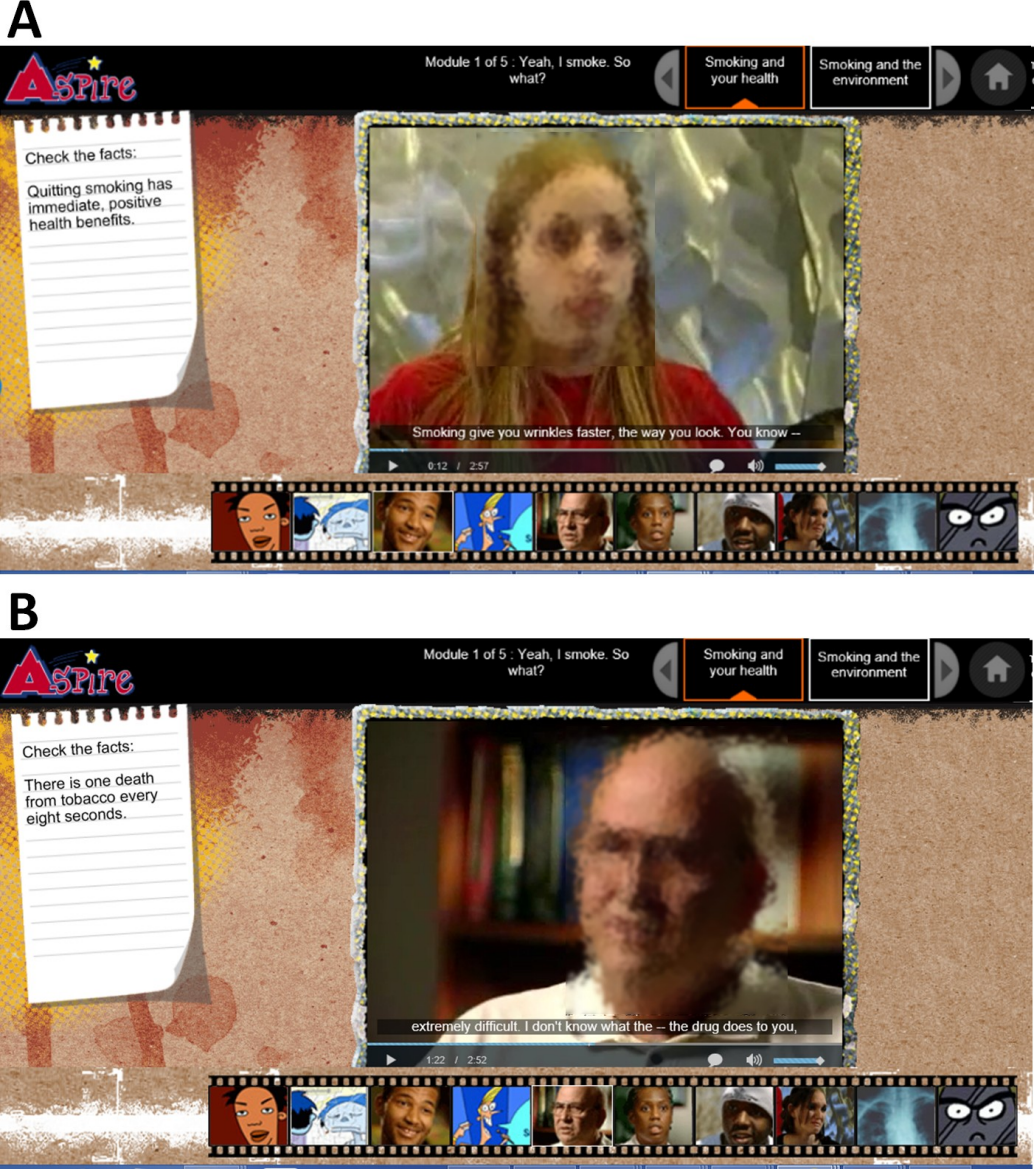
Examples of videos in ASPIRE. In this figure, (a) adolescents and (b) adults are sharing their experience with smoking. These videos are part of the ASPIRE material. They do not present participants in the study. Faces have been censored.

Tailoring, an important feature of ASPIRE, emerged when participants were asked what they liked most about the program and what to add to the program to make it more successful. Adolescents preferred ASPIRE content that is tailored to their demographics. Mainly, they expressed similarities between the characters and themselves. They felt that they can “relate” (Male) better to people their age who discuss the effects of smoking and explain the reasons not to smoke. On the other hand, adolescents tended to express disinterest in ASPIRE videos that had adults lecturing about smoking prevention. This report was common among adolescents, with the exception of one participant who expressed liking a video that included an adult celebrity athlete because of his interest in skateboarding: “There was the one [video] about Tony Hawk with skateboarding. I liked that one because I skateboard” (Male). Fig 4 presents examples of testimonies from young teens and adults sharing their experience with smoking. Some reports of tailoring include:

*-*“*I think that the ones with kids in them are most enjoyable for me because*, *you know*, *they’re my age*, *they’re talking about it*, *and I can get what they’re saying*. *The most boring videos were like testimonies from adults*, *because you know they’re not talking on the same plane as children my age” (Male)*.

*-*“*[Add] kids talking, like teenagers*. *[Add] more teenagers talking about how they don’t smoke*. *Because there are adults there (in ASPIRE), but some kids don’t think adults appeal to them*.” *(Female)*

#### Preference for interactivity

Adolescents expressed preference for interactivity over entertainment. They believed that interactivity could get them more involved in the content and allow them to discover smoking-related information:

-*“[What I like most about ASPIRE is] the interactive things to get you more involved instead of just watching videos” (Male)*

-*“[I like] how you can interact with the website* … *In the interactive stuff you could click on something and information will pop up*.*” (Male)*

**Fig 5:**
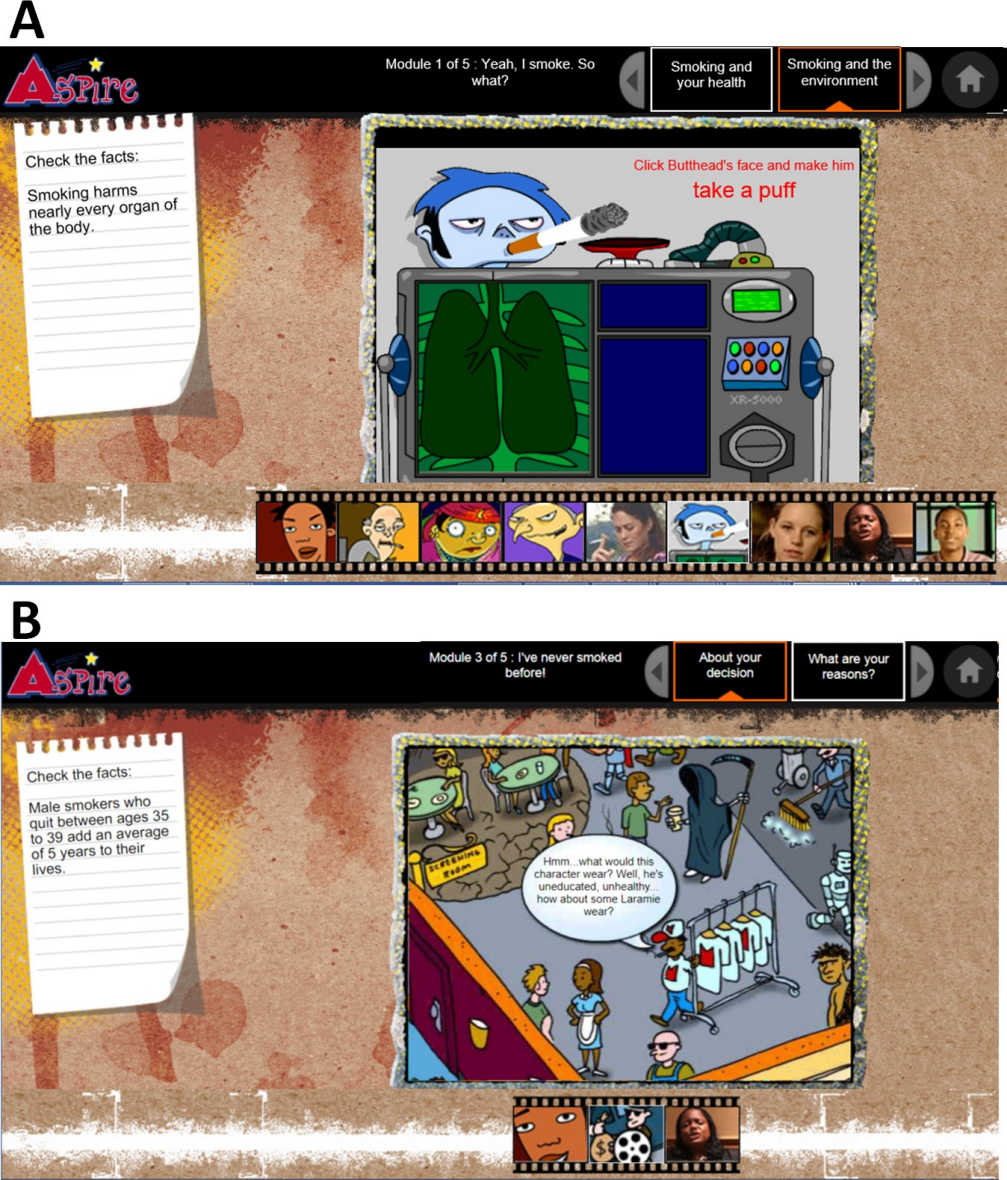
Examples of interactive activities that induce learning through exploration.

Some adolescents presented their Web-based experience by describing the exploration of “different scenarios” as a result of interactivity. In particular, their detailed description clarified their interest in real-time response of input and output, and explained that it would be desirable to have more available choices for ASPIRE users. Fig 5 presents activities that make use of exploration for learning. One example is an activity that informs about the chemical content of cigarettes:

-*“[The most appealing activity is] the one where you can click on it and it shows you different scenarios*, *and different things that inflict you from smoking*. *It completely listed all the effects and all the ingredients of it*, *and what those ingredients have*, *and where those ingredients can be found around you*.*” (Male)*

#### Suggestions for future design

Adolescents conveyed a need for game-based activities in ASPIRE: “[I would] make games on the website” (Male) and “I would add more of the activities. I would put games in there” (Male). Adolescents also shared creative ideas for smoking prevention games. As they explained, such ideas were mainly based on their prior experience with online fast-paced and simple games.

-*“Let’s say there are fast objects falling and you have to choose the good ones from the bad ones*. *Say if an apple is falling*, *it is what you want to get but if a cigarette is falling or a jar of tar*, *you have to avoid*. *You have to grab the good ones and avoid the bad ones*. *Also*, *you know the game temple run*? *You can avoid the bad things in the game and try to get the good things… You’re like in control of your life if you don’t choose the wrong path*. *[If you choose the wrong path] then*, *you face the consequences*.*” (Male)*

In addition to game-based activities, adolescents believed that they could benefit from social interactivity (i.e., interactivity between individuals, as opposed to interactivity between technologies and users). In ASPIRE, videos made adolescents feel that ASPIRE is stagnant and lengthy in content, and they perceived time as passing more slowly. This may be due to the unidirectional nature of this communication channel and the lack of user involvement through interactivity.

-*“[I want to have discussions] because you would talk to people and discuss what you learned*, *and that’s a lot of stuff*. *I mean [it] wouldn’t be just sitting there*. *Well I like watching videos but not for three hours*, *because it gets uncomfortable*.*” (Female)*

### Major theme II: Psychological and health outcomes of ASPIRE

#### Emotional outcomes

In addition to their description of their experience with ASPIRE content, adolescents tended to report psychological outcomes of enjoyment involving moments of positive and negative emotions. In particular, although succinct, adolescents reported surprising health facts, disgusting graphics, and humorous videos: “[I liked] probably the cartoons. They were funny and they were cute to watch” (Female), “[The graphic videos], that’s just nasty and I don’t want to look like that” (Female), “The gross pictures… they were disgusting” (Female), and “It’s surprising how it (smoking) gets you sick in the lungs… People that don’t smoke still get sick with people who smoke. Like if you smoke and I don’t smoke, I still get lung cancer.” (Male)

#### Shift in attitudes and beliefs

As a result of the ASPIRE experience, adolescents were provoked to think further about the effects of smoking and shifted their attitudes and beliefs. Although not very articulate, adolescents reported that ASPIRE changed their perspective and made them more aware of the consequences of tobacco use. These reports indicate that adolescents moved toward the contemplation stage of the TTM, with statements such as “opened my eyes” and “made me more aware”, and they are considering preventative action against tobacco use through their unwillingness to smoke (a thematic category presented below). In addition, such statements of contemplation indicate adolescents’ perception of tobacco risks, which is a potent predictor of tobacco use [31].

-*“… Going through this program*, *it opened my eyes to see how bad smoking is” (Male)*

-*“It (ASPIRE) made me more aware about the effects of smoking*.*” (Male)*

Adolescents did not only report a simple shift in beliefs at a cross-sectional level, but also tended to express their change in perception over time. For instance, two adolescents spoke about how their perspective has changed from before to after the ASPIRE experience. Their reports show evidence of improved awareness and the perception of the severity of tobacco use. Their report also highlight a shift in perceived social norms. Prior to the ASPIRE program, they believed that smoking is a relatively safe behavior that is only engaged by adults. It is through ASPIRE that they learned how others their age use tobacco, how they can become tempted, and the negative consequences of the behavior.

-*“… When I was younger*, *I thought smoking was something grownups do*. *Now I see it is just bad and nasty*. *It (ASPIRE) changed me…” (Female)*

-*“I have changed my perspective*. *I used to think it is normal and not that bad*, *but now I do know how bad it is*.*” (Male)*

#### Unwillingness to smoke

As a logical next step during the interviews, it was common for adolescents to express their lack of intention to smoke and mention health information related to the negative effects of smoking. Going beyond a single expression against smoking, adolescents elaborated on the health consequences of smoking such as lung cancer, addiction, and having yellow teeth. By pointing out some videos in ASPIRE (e.g., a video with graphics of cancer patients, a humoristic video about a smoker, and a celebrity sharing her experience with second-hand smoking), adolescents recalled symptoms of smoking:

-*“I don’t want to smoke cigarettes because I don’t want to get [like] addicted*, *and like all those faces on the video*. *That’s just nasty and I don’t want to look like that*. *I want to be healthy*.*” (Female)*

### Major Theme III: social interaction outcomes of ASPIRE

#### Social accountability

Adolescents explained that others should be accountable for their actions when they smoke. Adolescents expressed others’ accountability by mentioning that the effects of smoking can affect others in the social network, even those who do not smoke. Their awareness of second-hand smoking was a crucial factor that drove them to hold smokers accountable for their actions. One participant in ASPIRE stated directly that “… it (smoking) can affect anybody and not just the person that smokes. It’s not just that one person, but everybody around you” (Male). Another adolescent highlighted that the ASPIRE experience: “… really made me not wanting the people around me to be doing it” (Male).

#### Interpersonal discussions and persuasion

Adolescents did not only feel that others should be responsible for their smoking behavior, but they also reported enthusiasm to initiate positive interpersonal conversations against smoking. In particular, participants reported that the website motivated them to (1) speak with others about the effects of smoking and (2) attempt to convince others to quit smoking. One adolescent reported: “… I would talk to people that I know who smoke and try to convince them not to smoke.” (Male)

The attempt to persuade others that they should not smoke went beyond verbal communication. For instance, one participant reported wanting to replay ASPIRE and show the program to others their age. Another participant planned a potential dialogue with a family member who smokes, attempting to create a persuasive message that she can use. Physical engagement and strategy-building in order to promote cessation among loved ones was prominent during the interviews. For instance, some adolescents shared:

-*“I may start talking to people about it*. *I have one friend who smokes but he doesn’t smoke cigarettes; he smokes electric cigarettes*, *and I may be talking to him about it*. *I may even show it (ASPIRE) to him*, *you know*, *if I can replay it*.*” (Male)*

-*“My grandma’ smokes*, *and I don’t want her to have yellow teeth and stuff like that*. *I want to tell her that she needs to stop smoking*. *When I see a pack of cigarettes I will say what is that for*? *And if she says for smoking*, *I will be like can I throw it away*?*” (Female)*

-*“I talked to my dad about smoking because he smokes a lot*. *I would tell him ‘it is really bad for you’*. *He smokes since before I was born*, *and he smokes like a pack a day*.*” (Female)*

## Discussion

Our qualitative findings are crucial for understanding ways to advance public health interventions for smoking prevention, such as ASPIRE. While the current version of ASPIRE is to some extent successful, there is potential for further improvement. In this study, adolescents reported on their experience with the intervention, psychological outcomes of their experience, and evidence for social interaction outcomes. Such findings highlight elements that are crucial for the update of the currently available ASPIRE program or the design of a novel interactive program. The introduction of the reported new components can potentially boost the success of ASPIRE.

Our participants gave importance to tailored material in ASPIRE. Tailoring, the presentation of information that is unique to user characteristics [32], is a design feature that can boost cognitive and behavioral outcomes [10, 33]. Tailoring content based on age was particularly important to adolescents. This finding is supportive of the E-ELM, which states that similarity to media characters can facilitate attitude change [18, 34]. While ASPIRE included tailoring based on age, future work can introduce a more sophisticated type of tailoring by presenting adolescents with information that is relevant to their gender, ethnic group, cultural preferences, and level of intention to smoke. The introduction of additional tailored material is supported by the evidenced success of tailored web-based interventions compared to nontailored approaches to health behavior change [35, 36].

Adolescents also reported preference for interactive activities over videos. In particular, they expressed interest in firsthand experience of health information through exploration and discovery. Such findings support the experiential learning theory (ELT), which states that individuals are able to learn through exploration [37, 38]. It is hence important that concentrate our intervention design on activities that are rich in interactive elements (e.g., clicking behavior, lists of information, different possible scenarios, and virtual environments) as well as tailored material, in order to facilitate learning and shift attitudes. Going beyond preference for interactivity, adolescents suggested a mix between entertainment and interactivity, using games. Unexpectedly, adolescents did not recommend the design of a full game-based intervention. Instead, they expressed interest in short quick games that resemble commonly known games in mobile phone applications (e.g., Temple Run) [39, 40]. Adolescents may prefer short games because they offer immediate feedback on actions and decisions, sometimes within the same interactive environment, allowing for an immersive experience, and helping to sustain interest in content [41]. As a result, adolescents may begin to understand the consequences of smoking through the gaming experience.

In addition to reporting design preferences, adolescents expressed social interaction outcomes that resulted from their experience in ASPIRE. In content, ASPIRE did not involve social interaction. The program was designed to provide medium interactivity (i.e., interaction between users and media content). Nevertheless, supportive of the E-ELM, the ASPIRE experience at the individual level may have motivated adolescents to engage in interpersonal discussions with family members and friends. Such discussions may amplify the effectiveness of interventions because they allow rehearsal and reinforcement of health information [18]. Discussions may also reduce stigma related to negative social norms concerning sensitive public health topics such as underage tobacco use [16, 42–44].

In the current study, some adolescents suggested the addition of social interaction elements to ASPIRE in order to amplify this outcome. As a result, we plan to introduce technology features that encourage connections between adolescents, allowing them to affirm their beliefs and foster new social networks that support a smoke-free environment. Counteracting peer pressure from smokers [43, 44], it is possible that adolescents who do not intend to smoke influence others who do want to smoke. In particular, interventions such as ASPIRE may allow adolescents to diffuse pro-health messages through positive social interactions. Such interactions can work to enhance the effectiveness of interventions.

Going beyond discussions, adolescents were motivated to persuade members of their social network to quit or avoid smoking. During the interviews, some adolescents began to design persuasive messages that they can apply with loved ones. For instance, one adolescent constructed an imaginary dialogue with her grandmother in order to convince her to quit smoking. While previous research has considered interpersonal discussion as an outcome of health interventions [14, 45, 46], none have considered interpersonal persuasion, which may create a successful strategy for the dissemination of health messages against smoking. Future work on public health interventions may consider preparing users to engage in successful persuasive conversations with others in their social network. Consequently, with a longer follow-up period, researchers may explore interpersonal persuasion in order to understand its effect on reinforcement of health messages.

In addition, the findings revealed a novel concept in smoking prevention: social accountability. *Social accountability* involves being mindful that the negative effects of smoking form a social concern to a group of individuals, such as a community [47–49]. In particular, adolescents believed that others should be accountable for their actions when smoking. As a result, the topic of smoking shifted from being a personal to an interpersonal and social health concern. The concept of social accountability as a health message strategy has been extensively applied for smoking cessation, when holding tobacco companies accountable for their actions. Particularly, smoking cessation programs included messages describing how tobacco companies have knowingly worked to persuade smokers to use products without any regard to their health, a strategy termed tobacco industry denomalization [50]. In addition, smoking cessation programs targeting secondhand smoke have worked to hold smokers accountable for their actions around their family members or during pregnancy [51, 52]. However, this is its first report as an outcome of an intervention, holding individual smokers accountable for their behavior. Future intervention design may consider introducing health messages that strengthen social accountability and allow adolescents to feel empowered to take actions in their community. As a result of ASPIRE, adolescents may become ambassadors who are ready to engage firsthand in anti-tobacco campaigns through a community-based participatory approach [53]. Additionally, future research may work to develop a scale that measures perceived social accountability in order to examine its change over time as a result of such interventions.

Finally, with respect to content related to tobacco, ASPIRE mainly discusses combustible products such as cigarettes, cigars, and hookah. However, one adolescent mentioned having friends who use electronic cigarettes. Current research indicates that nearly half of adolescents and young adults who use tobacco tend to engage in both smoking and vaping [54, 55]. This is particularly a problem among adolescents, considering that their initial use of vaping products has been linked to their subsequent cigarette smoking [56]. While vaping products are not particularly addressed in this version of ASPIRE, we plan to discuss them and their potential risks to youths in a future program.

### Strengths and limitations

The strength of this study is that it provided an opportunity to explore the thoughts of adolescents with regard to an evidence-based smoking prevention program. While ASPIRE has been documented to be successful, adolescents made it clear that several aspects of the program may need to be updated. It is also one of the first manuscripts to uncover new outcomes from Web-based smoking prevention interventions (i.e., interpersonal persuasion and social accountability). Such outcomes form novel concepts that deserve further conceptualization and means of measurement in future studies. The development of a codebook was useful in the qualitative analysis since it helped to identify new themes and allowed coders to reach 100% agreement. One limitation of this study was that this sample of adolescents was randomly drawn from the experimental group of the ASPIRE Reactions trial. Nevertheless, the objective was to randomly select the sample in order to perform the exit interviews. Compared to random sampling, a purposive sample of similar demographic characteristics to the sample in the trial may have been a better option to increase the likelihood that the feedback represents the participants of the original program. Unfortunately, the selection process had to abide by the ethical request of the after-school programs, whereby the full demographic characteristics of interview participants could not be collected. As a result, specific individuals could not be selected for participation. Nevertheless, gender information was collected, and it was found to be similar to that of the original sample (61% male in the original sample, and 65% male in the interview sample). To have only twenty interviews conducted is normal in the context of qualitative research, and we acknowledge that the reports may not be statistically representative of the entire adolescent population. Previous research on intervention design has indicated that thematic saturation with adolescents can be reached by 20 interviews [57, 58]. The qualitative subsample only included participants randomized to ASPIRE. While, adolescents in control group were not interviewed, it is unclear to what extent some of the viewpoints of the participants would be true more generally or whether they are a feature of the intervention subsample. Our interviews are only the first step toward designing novel health communication features that can improve smoking prevention programs such as ASPIRE. As a result of the current findings, we plan to (1) improve the user experience, (2) further explore the nature of social outcomes of smoking prevention programs (e.g., social accountability and interpersonal persuasion) and (3) develop and test measures of these outcomes. This line of research can reveal new theoretical processes that result from smoking prevention interventions.

## Conclusions

In conclusion, this qualitative study provided valuable insight following a randomized controlled trial for a smoking prevention intervention (ASPIRE), regarding adolescents’ subjective experience with the program, suggestions for improvement in content, and report of individual and social outcomes. We anticipate that the results of this study will allow us to develop a novel smoking prevention intervention with more advanced features (e.g., interactivity and tailoring) that can promote social interaction. The findings are relevant to the public health mission of acquiring new knowledge to develop, test, and translate prevention interventions that target the initiation of tobacco.

## Supporting information

S1 File COREQ checklist. (DOCX)

S2 File Interview questions. (DOCX)

S3 File Analysis codebook. (DOCX)

S4 File Qualitative data. (DOCX)
