# Supplementary material for: Revealing users’ experience and social interaction outcomes following a web-based smoking prevention intervention for adolescents: A qualitative study
Source: PLoS One. 2019 Oct 17;14(10):e0223836. doi: 10.1371/journal.pone.0223836 (PMC6797109; doi:10.1371/journal.pone.0223836)
Supplement: S1 File — (DOCX) [file pone.0223836.s001.docx]

**Supplementary File 1:** COREQ checklist

| **Items** | **Information** | **Location in manuscript** |
| --- | --- | --- |
| **Domain 1: Research team and reflexivity** |  |  |
| **Personal Characteristics** |  |  |
| 1. Interviewer/facilitator: Which author/s conducted the interview or focus group? | GEK and a trained research assistant conducted the interviews. The research assistant is not an author on this manuscript. | Materials and Methods, Data Collection, page 9, line 184 |
| 2. Credentials:  What were the researcher’s credentials? E.g. PhD, MD | The researchers on this project had the following credentials: B.Sc., M.D., Dr.PH, and Ph.D. The interviewers’ credentials were B.Sc., and Ph.D. respectively | Title page, page 1 and Materials and Methods, Data Collection, page 9, line 184. |
| 3. Occupation: What was their occupation at the time of the study? | Postdoctoral Fellow and Research Assistant | Materials and Methods, Data Collection, page 9, line 184 |
| 4. Gender: Was the researcher male or female? | Interviewers were one male and one female | Materials and Methods, Data Collection, page 9, line 184 |
| 5. Experience and training: What experience or training did the researcher have? | At the time of the interviews, researchers had a full training in the principles of qualitative research. | Materials and Methods, Data Collection, page 9, line 185 |
| **Relationship with participants** |  |  |
| 6. Relationship established: Was a relationship established prior to study commencement? | Yes. Prior to the interviews, a relationship of one month was established with the participants in order to build trust and freedom in information-sharing. This relationship was established through the randomized trial that was conducted prior to the interviews. The interviewers were present during the trial data collection. In addition, the interviewers volunteered as staff members in the after-school program. Such volunteering included engaging in summer camp activities with the adolescents. | Materials and Methods, Data Collection, page 9, lines 190-193 |
| 7. Participant knowledge of the interviewer: What did the participants know about the researcher? e.g. personal goals, reasons for doing the research | Participants were briefed on the purpose of the study and understood that it was a research project on their experience with the website ASPIRE. | Materials and Methods, Data Collection, page 9, lines 195 |
| 8. Interviewer characteristics: What characteristics were reported about the interviewer/facilitator? e.g. Bias, assumptions, reasons and interests in the research | There were no interviewer-related biases identified in this study. | Materials and Methods, Data Collection, page 9, lines 187 |
| **Domain 2: study design** |  |  |
| **Theoretical framework** |  |  |
| 9. Methodological orientation and Theory: What methodological orientation was stated to underpin the study? e.g. grounded theory, discourse analysis, ethnography, phenomenology, content analysis | In this study, a codebook was first developed based on expectations from the extended-elaboration likelihood model. Then, open coding with thematic content analysis was conducted in order to identify new themes. Following grounded theory, as new themes emerged, the codebook continued to evolve, as part of an iterative, inductive process. This process continued until thematic saturation was reached. Supportive of the grounded theory, retrieved themes are deemed appropriate as they provide new concepts that extend the E-ELM. | Materials and Methods, Qualitative Analysis, page 10, lines 208-216 |
| **Participant selection** |  |  |
| 10. Sampling: How were participants selected? e.g. purposive, convenience, consecutive, snowball | For the current study, participants from the ASPIRE condition were randomly selected to take part in an exit interview upon completion of the trial. | Materials and Methods, Recruitment and Sampling Strategy, page 7, line 150 |
| 11. Method of approach: How were participants approached? e.g. face-to-face, telephone, mail, email | Face-to-face | Materials and Methods, Recruitment and Sampling Strategy, page 7, line 152 |
| 12. Sample size: How many participants were in the study? | Twenty | Results, page 11, line 233 |
| 13. Non-participation: How many people refused to participate or dropped out? Reasons? | None of the approached adolescents refused to participate or dropped out. | Materials and Methods section, under Data Collection, page 9, lines 190-194 |

| **Setting** |  |  |
| --- | --- | --- |
| 14. Setting of data collection: Where was the data collected? e.g. home, clinic, workplace | In this study, interviews were conducted following a randomized controlled trial titled ASPIRE Reactions (Registered at ClinicalTrials with the registration number: NCT02469779). At each after-school program, participants entered the interview room individually and were seated comfortably in a living room setting. | Materials and Methods section, under Data Collection, page 9, lines 196 |
| 15. Presence of non-participants: Was anyone else present besides the participants and researchers? | No | Materials and Methods section, under Data Collection, page 9, lines 196 |
| 16. Description of sample: What are the important characteristics of the sample? e.g. demographic data, date | Age ranged from 11 to 18 years; Out of respect to the wishes of the after-school programs, only gender information was obtained during the interviews. Participants were 7 females and 13 males. | Results, page 11, line 234 |
| **Data collection** |  |  |
| 17. Interview guide: Were questions, prompts, guides provided by the authors? Was it pilot tested? | The interview instrument was pilot-tested with 10 adolescents, to reach a final set of 11 interview questions. The questions are provided in a supporting information file | Materials and Methods, Interview Instrument, page 8, line 176 |
| 18. Repeat interviews: Were repeat interviews carried out? If yes, how many? | No | Materials and Methods, Data Collection, page 10, line 205 |
| 19. Audio/visual recording: Did the research use audio or visual recording to collect the data? | The interviews were audio recorded using a handheld audio-recorder. | Materials and Methods, Data Collection, page 9, line 189 |
| 20. Field notes: Were field notes made during and/or after the interview or focus group? | Yes, additional field notes were made. | Materials and Methods, Data Collection, page 10, line 204 |
| 21. Duration: What was the duration of the interviews or focus group? | The interview lasted approximately 15 minutes in length. | Materials and Methods, Data Collection, page 10, line 203 |
| 22. Data saturation: Was data saturation discussed? | Yes, coding continued until thematic saturation was reached. | Materials and Methods, Qualitative Analysis, page 10, line 223 |
| 23. Transcripts returned: Were transcripts returned to participants for comment and/or correction? | No | Materials and Methods, Qualitative Analysis, page 11, line 230 |
| **Domain 3: analysis and findings** |  |  |
| **Data analysis** |  |  |
| 24. Number of data coders: How many data coders coded the data? | Two | Materials and Methods, Qualitative Analysis, page 10, line 211 |
| 25. Description of the coding tree: Did authors provide a description of the coding tree? | Open coding with thematic analysis and the grounded theory approach. Coding was described in methods section, and a codebook is provided as a supplementary file. | S2 File and Materials and Methods, Qualitative Analysis, page 10, line 208-230 |
| 26. Derivation of themes: Were themes identified in advance or derived from the data? | Some themes were derived in advance, and then additional themes were derived from the data | Materials and Methods, Qualitative Analysis, page 10, line 208-230 |
| 27. Software What software, if applicable, was used to manage the data? | Microsoft Word and Excel | Materials and Methods, Qualitative Analysis, page 11, line 230 |
| 28. Participant checking: Did participants provide feedback on the findings? | No | Materials and Methods, Qualitative Analysis, page 11, line 229 |
| **Reporting** |  |  |
| 29. Quotations presented: Were participant quotations presented to illustrate the themes / findings? Was each quotation identified? e.g. participant number | Yes, specific comments were supported with direct quotes attributed to anonymized participants by gender | Results, page 13-19, lines 250-392 |
| 30. Data and findings consistent: Was there consistency between the data presented and the findings? | Yes | Results, page 13-19, lines 250-392 |
| 31. Clarity of major themes: Were major themes clearly presented in the findings? | Yes, major themes were presented as subtitles in the Results section. | Results, page 13-19, lines 250-392 |
| 32. Clarity of minor themes: Is there a description of diverse cases or discussion of minor themes? | Minor themes are discussed in the manuscript and reported in full. | Results, page 13-19, lines 250-392 |
