# Supplementary material for: Revealing users’ experience and social interaction outcomes following a web-based smoking prevention intervention for adolescents: A qualitative study
Source: PLoS One. 2019 Oct 17;14(10):e0223836. doi: 10.1371/journal.pone.0223836 (PMC6797109; doi:10.1371/journal.pone.0223836)
Supplement: S2 File — (DOCX) [file pone.0223836.s002.docx]

**Supplementary Material 1**

**Interview Questions:**

- Overall, what did you think of ASPIRE?
- Which video did you like most in ASPIRE?
- What did you like most about this video?
- Which video did you like least in ASPIRE?
- What did you like least about this video?
- Which activity did you like most in ASPIRE?
- What did you like most about this activity?
- Which activity did you like least in ASPIRE?
- What did you like least about this activity?
- If you were given all the resources possible, and were asked to make ASPIRE better, what would you change, add, or remove? Tell me more.
- What did ASPIRE make you want to do, that you would not have done otherwise?
