# Supplementary material for: Revealing users’ experience and social interaction outcomes following a web-based smoking prevention intervention for adolescents: A qualitative study
Source: PLoS One. 2019 Oct 17;14(10):e0223836. doi: 10.1371/journal.pone.0223836 (PMC6797109; doi:10.1371/journal.pone.0223836)
Supplement: S3 File — (DOCX) [file pone.0223836.s003.docx]

**Supplementary Material 2**

**Codebook**

*The theoretical framework and topic that are inspected through the codebook are the E-ELM and media feature preferences*

**Central Processing:**

***Health information.***

Expression of low or no intention to smoke?

1 Yes – 0 No; How many times?

[Example: **I don’t want to smoke cigarettes**]

[Example of counting number of times:

**I don’t want to smoke cigarettes** because I don’t want to get addicted, and like all those faces on the video. That’s just nasty and I don’t want to look like that. **I want to be healthy.** [The most boring videos were] the one with the students and the video with the first doctor. But overall it was a good program, preventing people not to smoke and to be aware of how smoke can affect your life in the future. [My favorite videos were] the testimonies.

- **2 times** ]

Report of health facts related to smoking prevention?

1 Yes – 0 No; How many times?

[Example: I don’t want to smoke cigarettes because **I don’t want to get addicted**]

Mention of knowledge gain?

1 Yes – 0 No; How many times?

[Example: I **learned**… ASPIRE **taught** me…]

***Preference for specific media features.***

Mention of audience-based entertainment (e.g., narrative, music, and video)?

1 Yes – 0 No; How many times?

[Definition: Allows individuals to act as spectators or audience members and witness an entertainment activity taking place]

[Example: The videos were my favorite, when they were talking about **the blue guy** and the date.]

Mention of modality?

1 Yes – 0 No

[Definition: mode of delivery, such as video, activity, cartoon, music, etc.]

[Example: And you know it lets me open my eyes. Some of the **videos**, because they were played a number of times.]

If yes, the modality was:

Video?

1 Yes – 0 No/No modality mentioned

Audio?

1 Yes – 0 No/ No modality mentioned

Mention of storyline?

1 Yes – 0 No; How many times?

[Example: Then you have people from the real world, you can actually share their **story** and **how hard it was for them to quit, how they quit and how they celebrate quitting and how they got to do the slipping and all that**.]

Was the narrative genre:

Drama?

1 Yes – 0 No/No narrative mentioned

Comedy/Humor?

1 Yes – 0 No/No narrative mentioned

Horror?

1 Yes – 0 No/No narrative mentioned

Mention of entertaining sound?

1 Yes – 0 No; How many times?

If yes, was it music?

1 Yes – 0 No; How many times?

If yes, was the music a

A melody?

1 Yes – 0 No; How many times?

A song?

1 Yes – 0 No; How many times?

If yes, was it a sound effect?

1 Yes – 0 No; How many times?

Mention of pure interactivity (e.g., clicking behavior, search for information, two-way interactions with interface)?

1 Yes – 0 No; How many times?

[Definition: involve an active interactivity with the medium without the presence of entertainment]

Mention of speed (i.e., real-time responses of input and output)?

1 Yes – 0 No; How many times?

[Example: The activity where you have to **click and click and click and nothing special happens. If you want to click, you need to click!** I learned a lot about smoking.]

Mention of wide range (i.e., large number of choices available to the user at a specific point in time)?

1 Yes – 0 No; How many times?

[Example: The one where you can click on it and it shows you different scenarios, and different things that inflict you from smoking. It completely listed all the effects and all the ingredients of it, and what those ingredients have, and where those ingredients can be found around you.]

Mention of Frequency (i.e., the number of times one is able to interact within an interface)?

1 Yes – 0 No; How many times?

Mention of mapping (i.e., similarity of a mediated platform with the physical real environment)?

1 Yes – 0 No; How many times?

[Example: …have people from the real world…]

Mention of Multimodality (i.e., information in various media formats)?

1 Yes – 0 No; How many times?

Mention of tailoring (i.e., information targeting users’ demographics)?

1 Yes – 0 No; How many times?

Mention of performance-based entertainment (e.g., activity with entertainment, fun activity)?

1 Yes – 0 No; How many times?

[Definition: A merging interactivity with entertainment that allows individuals to participate firsthand in the entertaining activity]

[Example: The interactive activity that I liked is the one where it gives you reasons why you would want to smoke and is it your mood or stress or things like that. It had a bunch of boxes with different categories of reasons.]

Mention of an interactive narrative (i.e., narrative modified by the user)?

1 Yes – 0 No; How many times?

[Example: When I clicked on it, it gave me different scenarios.]

Mention of exploration of virtual environments?

1 Yes – 0 No; How many times?

[Example: The one where you can click on it and it shows you different scenarios, and different things that inflict you from smoking. It completely listed all the effects and all the ingredients of it, and what those ingredients have, and where those ingredients can be found around you.

Find the little film things. You know like where you had to find the cigarette in the movie casting and all that. That was difficult that’s all. It was hard to find. I had a hard time trying to find it.]

Mention of a gamified activity? (i.e., use of game elements in the context of non-gaming platforms)

1 Yes – 0 No; How many times?

**Emotional Involvement**

Mention of an emotional state (e.g., happy, sad, angry, surprised, excited, disgusted)?

1 Yes – 0 No; How many times?

[Definition: Use of adjectives that describe emotions]

[Example: It was surprising to see how much secondhand smoking can be harmful.]

**Attitude Change and Thought Provocation:**

Mention of change in perspective?

1 Yes – 0 No; How many times?

[Example: ASPIRE opened my eyes; changes my perspective]

**Rehearsal and Positive Interpersonal Communication:**

Mention of social connections?

1 Yes – 0 No; How many times?

Mention of telling others about ASPIRE?

1 Yes – 0 No; How many times?

Mention of telling others about the effects of smoking?

1 Yes – 0 No; How many times?

**Users as Opinion Leaders:**

Mention of social accountability?

1 Yes – 0 No; How many times?

Mention of convincing others to quit?

1 Yes – 0 No; How many times?

**Implications for Future Design of ASPIRE:**

Mention what to add as audience-based entertainment?

1 Yes – 0 No

Add a video?

1 Yes – 0 No/No mention of what to add as audience-based entertainment

Add music?

1 Yes – 0 No/No mention of what to add as audience-based entertainment

Mention what to remove as audience-based entertainment?

1 Yes – 0 No

Remove video?

1 Yes – 0 No/No mention of what to remove as audience-based entertainment

Remove music?

1 Yes – 0 No/No mention of what to remove as audience-based entertainment

Mention what to add as pure interactivity?

1 Yes – 0 No

Mention what to remove as pure interactivity?

1 Yes – 0 No

Mention what to add as performance-based entertainment?

1 Yes – 0 No

Add games?

1 Yes – 0 No/No mention of what to add as performance-based entertainment

[Definition: including words such as game and play]

Mention ideas for games?

1 Yes – 0 No/No mention of what to add as performance-based entertainment

Add other kind of performance-based entertainment?

1 Yes – 0 No/No mention of what to add as audience-based entertainment

Mention what to remove as performance-based entertainment?

1 Yes – 0 No

Remove games?

1 Yes – 0 No/No mention of what to remove as performance-based entertainment

Remove other kind of performance-based entertainment?

1 Yes – 0 No/No mention of what to remove as audience-based entertainment

Mention to add social interactivity?

1 Yes – 0 No [Definition: activity involving interaction with others online or in person, as part of the e-Health experience]
