# Supplementary material for: Revealing users’ experience and social interaction outcomes following a web-based smoking prevention intervention for adolescents: A qualitative study
Source: PLoS One. 2019 Oct 17;14(10):e0223836. doi: 10.1371/journal.pone.0223836 (PMC6797109; doi:10.1371/journal.pone.0223836)
Supplement: S4 File — (DOCX) [file pone.0223836.s004.docx]

**1**

**Female**

What did you think of the program?

I don’t want to smoke cigarettes because I don’t want to get [like] addicted, and like all those faces on the video. That’s just nasty and I don’t want to look like that. I want to be healthy.

Which ones were the most boring videos to you?

[The most boring videos were] the one with the students and the video with the first doctor. But overall it was a good program, preventing people not to smoke and to be aware of how smoke can affect your life in the future.

Which ones were your favorite videos?

[My favorite videos were] the testimonies.

**2**

**Female**

How did you find aspire? First reaction?

I thought it was good. I thought it was something else, like full of questions and stuff. It ended up being like videos and cool stuff.

What did you like best about it?

That they had the funny cartoons.

What did you like least about it?

None. Nothing. I can’t think of anything.

When I was younger, I thought smoking was something grownups do. Now I see it is just bad and nasty. It changed me because I realized that my grandma’ smokes and I don’t want her to have yellow teeth and stuff like that. I want to tell her that she needs to stop smoking. When I see a pack of cigarettes I will say what is that for? And if she says for smoking, I will be like can I throw it away?

**3**

**Female**

What was your reaction to ASPIRE?

That was cool; how it tell you not to smoke and it is bad.

What was your favorite part in it?

The three monkeys. React, remember, and reach.

What was your most boring part in aspire?

When people were talking.

Do you have in mind something specific about the people talking?

Some videos of people were okay. But some people were telling you smoking was okay. Not that it’s totally okay, but that they do it. When they were talking about smoking, I skipped the video. I don’t smoke I don’t like people telling me that they do smoke.

From everything that you’ve learned what would you do now that you wouldn’t have done before?

Remember not to smoke.

**4**

**Male**

What did you think of ASPIRE?

It’s a good program. It really promotes not smoking.

What did you like most about it?

The interactive things to get you more involved instead of just watching videos.

Is there a specific interactive part that you remember you liked?

The one where you can click on it and it shows you different scenarios, and different things that inflict you from smoking.

How was it helpful?

It completely listed all the effects and all the ingredients of it, and what those ingredients have, and where those ingredients can be found around you.

The program also has different people talking about how they don’t like it and how it affected them. And you know it lets me open my eyes.

What was the least fun part about the program?

Some of the videos, because they were played a number of times. For example, there is the video with the comedian on there, and she talks about how she got sick from second hand smoke, and it was played three or four times. But other than that, everything else was good.

Did you feel like there was a video that was the most boring?

I think that the ones with kids in them are most enjoyable for me because, you know, they’re my age, they’re talking about it, and I can get what they’re saying. The most boring videos were like testimonies from adults, because you know they’re not talking on the same plane as children my age. They’re talking in a general sense, and the kids are talking more personal.

If you were given all the money in the world and you were going to make this better what would you add to it?

I would add more activities; maybe add some songs about it and put it on there, and something to make it more interactive.

What would you take out of it?

Well, you know, my dad smokes and I don’t like it. I always tell him about it but going through this program, it opened my eyes to see how bad smoking is. I won’t ever try it.

How do you feel it is going to make you react after this? What is it going to make you do now that you wouldn’t do before?

I may start talking to people about it. I have one friend who smokes but he doesn’t smoke cigarettes; he smokes electric cigarettes, and I may be talking to him about it. I may even show it to him, you know, if I can replay it.

**5**

**Male**

What is your reaction on this?

The website is good, because you know, I learned about things in smoking, and I don’t want to smoke.

What did you like most about the website?

The videos were my favorite, when they were talking about the blue guy and the date.

What was your least favorite video?

The activity where you have to click and click and click and nothing special happens. If you want to click, you need to click!

What would you do now that you wouldn’t have done before?

I learned a lot about smoking. I don’t like to smoke anyway.

I have changed my perspective. I used to think it is normal and not that bad, but now I do know how bad it is.

**6**

**Male**

What did you think of ASPIRE?

It was kind of long.

What did you like most about it?

The funny videos and how you can interact with the website. The videos were like joking around. In the interactive stuff you could click on something and information will pop up.

What was most boring about the program?

It was kind of long.

What’s the least fun video?

I don’t remember.

**7**

**Male**

What was your impression of ASPIRE?

It gave a lot of information; I didn’t really know most of it, and it changed my opinion. I already been against smoking, but it really made me not wanting the people around me to be doing it. I realized the health effects it could give.

What did you like most about the website?

The interaction. It wasn’t just me watching a video; I could click and give my opinion and things. It felt more personalized, like for me not just for everybody. The interactive activity that I liked is the one where it gives you reasons why you would want to smoke and is it your mood or stress or things like that. It had a bunch of boxes with different categories of reasons.

What was the least appealing part of ASPIRE?

It was one video, the one of the comedian, and I think it’s because it was in there twice, a five-minute long video. It was a comedian lady talking about second-hand smoke.

Is there a specific video that you liked more than others?

I think it was the one where they were talking about slipping. And it was all those people talking about how they slipped and giving their different ways to go past that.

What about a video that you really felt was boring?

I can’t think of one that stood out as being really boring.

I think it was the one that was talking about the blue guy and it was talking about chemicals, and that one didn’t really stand out until the end, when it was talking about what exactly those chemicals are and all the things in them, and I didn’t pay attention to any of that.

What activity was not fun?

The one that shows a brain and you had to click it and flush out a chain of thought; that activity, I kind of got stuck on it for a second and I didn’t really get that one.

What would you add to this website?

I would add kind of like a finisher at the end; because at the end it would have been better with an activity instead of like a test and repeating one of the videos. It could have actually brought it all together.

What would you take out?

I can’t think of anything that I would take out.

**8**

**Male**

What did you like best in ASPIRE?

Probably like stuff when you are not watching videos; like clicking on stuff.

What was your least favorite part in ASPIRE?

Continuing to watch video nonstop

Which video was the most boring?

Probably the cartoons

If you were given all the money in the world and you had to change something in ASPIRE what would it be?

Make games on the website.

What would you take out of the website to make it better?

Some of the videos

Which videos did you like?

The humans talking. The cartoons talking were too cheesy.

What was your favorite interactive part in ASPIRE?

When clicking on the stuff and it would tell you about it.

Which interactive activity? All of them.

**9**

**Male**

What did you like most about ASPIRE?

It was interactive and you can relate to it a lot. Like with the activities, a lot of teenagers can understand it; it is not hard to understand.

What was your favorite part in it?

Clicking on stuff, like clicking in the activities on things to find out more about smoking. I remember clicking on people smoking and it tells you how you can get help from family members and your community.

What was your least favorite part in aspire?

Sometimes the videos repeated, so I don’t want to watch the same video again.

What video was your most boring?

I don’t remember.

Is there a video you liked?

There was the one about Tony hawk with the skate boarding. I liked that one because I skateboard.

What is the second video you liked?

The video with the comedian because she got lung cancer even though she did not smoke. She was funny.

What would you add to aspire?

I would add more of the activities. I would put games in there.

What would you change in aspire?

Sometimes they were repeating the same thing, about how bad smoking was and the bad effects of smoking so I would take away some of that.

How did you feel aspire affected you?

It made me more aware about the effects of smoking and especially second-hand smoking; how it can affect anybody and not just the person that smokes. It’s not just that one person, but everybody around you. I would talk to people that I know who smoke and try to convince them not to smoke.

**10**

**Male**

What did you think of it?

It was really informative and had a lot of information.

What did you like most about it?

The activities you get to do.

Which one was your favorite?

Where they would give you a situation, and how you would react to the situation; like certain types of situations rather than you smoking a cigarette; like the alternatives.

What did you like least about it?

Some of the videos were really long.

Can you tell me about a video that you didn’t like?

It was during module 1 and one of the first few videos, she kept repeating the same thing over and over again. If I were in the video I would have cut her off. It was a young person.

You said you liked the activities and you liked the scenarios; Is there another one you liked?

The one that shows the solar system and it shows the people what life can give to you.

What is your least favorite activity?

The fortune teller one; It was back in module 1. I felt that it was too cheesy. You had to click on something and it would tell you your fortune.

What was your least favorite video?

It is the one I told you. The one of module 1.

What was your most favorite video?

The one about the doctor that talked about how he can help adolescents with their problems with something or anything else. I thought that was really cool; that you can like help people.

If you were given a lot of money and were asked if you could add something to aspire what would you add?

Shorter, but more attention-grabing videos. Kind of like the ones they show in health class. Back when I was in health class, my teacher would show us a before and after of people who have been through those lapses of smoking and trying to quit, and there is one video of one who had surgery on their face and I guess that interview has more impact on the viewer.

What did you think of the graphics that we showed in ASPIRE?

They weren’t too bad.

And what else would you add?

[I would add] more activities, games; matching the situation with the solution, like a match game. Say it gives like a solution and there is a multiple choice of solutions and you have to fill in the blank. Say there is a solution and a colon. You have to drag the solution past the colon. Also shooting games. Flashplayer games online can be used. Like a fast-paced game. Let’s say there are fast objects falling and you have to choose the good ones from the bad ones. Say if an apple is falling, it is what you want to get but if a cigarette is falling or a jar of tar, you have to avoid. You have to grab the good ones and avoid the bad ones. Also, you know the game temple run? You can avoid the bad things in the game and try to get the good things.

What does that teach you?

You’re like in control of your life if you don’t choose the wrong path.

What if you choose the wrong path?

Then, you face the consequences, you become slower.

**11**

**Male**

Tell me what you thought of your interaction with this website?

It’s surprising how it (smoking) gets you sick in the lungs.

It has good videos and stuff like that. People that don’t smoke still get sick with people who smoke. Like if you smoke and I don’t smoke, I still get lung cancer.

Which video was your favorite?

The one where you pick people that smoke, and it makes noise. The one with Hollywood.

The most boring is the video where people talk.

The one with the globe and it tells you the future and stuff like that when you smoke. I liked that one.

After going through ASPIRE do you feel like it will make you do something you haven’t done before?

Yes. It made me learn what smoking does to you and stuff like that.

What is ASPIRE going to make you do?

I learn more information. That’s it.

If you were given a lot of money to fix this website, what would you do to it?

Make it have more activities.

If you were going to take out a part of ASPIRE, what would it be?

The videos when people talk. When the doctors talk.

Which one was the most boring activity?

The one where there is a person that smokes and you have to pick the answer like what kind of chemicals they are smoking. All this stuff they add inside of it like paint, and rat poison; I didn’t like that.

Was it a boring activity?

Yes

How would you fix it to make it more interesting?

Nothing in mind.

**12**

**Male**

What did you think of the program?

It was alright. It gave me information about smoking. I learned facts about smoking and what it can do to you.

What was one of the things that you learned?

That second-hand smoke can kill you.

What was your favorite video in ASPIRE?

The one on the second-hand smoking. It was a real video.

What was in the video that taught you about the second-hand smoke?

It showed pictures of people, like what happened to them after the second-hand smoke. Like some people had their face gone. It showed that second-hand smoke can really kill you.

What was your least favorite video?

Some of the videos I didn’t like. They were long.

If you had all the money in the world to fix the program what would you do to it?

I would make it shorter.

I preferred the cartoons. They were funny.

**13**

**Female**

What did you think of ASPIRE?

It was cool. It told me a lot about smoking and the bad effects of it.

What did you like most about it?

Probably the cartoons. They were funny and they were cute to watch.

What did you like least about ASPIRE?

The gross pictures, because they were disgusting.

What about the most boring thing about ASPIRE?

It probably have to do with the questions.

What questions?

The quizzes inside ASPIRE, but they taught me a lot.

What about your most boring activity in ASPIRE?

I actually don’t know. I just like to sit and watch things.

If you had all the money in the world and you were asked to make ASPIRE better what would you change in it? To change their vision of smoking.

Yes and there was stuff that was repeated. I didn’t like how it was repeated all the time. Like, kids talking like Teenagers. More teenagers talking about how they don’t smoke. Because there are adults there but some kids don’t think adults appeal to them.

What would you add to it?

I don’t know. Like a game they can play. More games and lesser videos. I do like watching videos but video games are better than adults talking.

If you had the opportunity to speak with friends and have discussions through ASPIRE with other people your age, would you like that and why?

Yes because you would talk to people and discuss what you learned, and that’s a lot of stuff. I mean wouldn’t be just sitting there. Well I like watching videos but not for three hours, because it gets uncomfortable.

Did ASPIRE make you want to do something that you wouldn’t have done without having gone through ASPIRE? What would that be?

Yes. I talked to my dad about smoking because he smokes a lot. I would tell him, it is really bad for you. He smokes since before I was born, and he smokes like a pack a day. My grandfather died from smoking.

**14**

**Female**

What did you think of ASPIRE?

I think it was a great website I really liked it. I liked how it has like the people talking about their opinions of smoking. Like in the videos, people talking about their experiences.

Which video was your favorite?

I don’t know. Like I don’t know. Like the first video with the brothers. Like the older brother is motivating the younger brother to stop.

What was the most boring video?

Probably like the one with the cartoon where he’s like in the restroom checking of his list.

Why is it the most boring?

Because like, it’s like predictable like, he’s like going through the list and nothing really exciting.

Of the videos where people talk, which ones were the most boring?

Probably like the one where like their experiences haven’t really gone anywhere, like they still have the habit of smoking and they’re not really trying to quit.

What about the activities? Did you like them?

They were okay. Some you have to click on to… I don’t know, they were ok.

Why? You were trying to tell me something.

It just like reads you what it says. I think that like you should have more information behind it. It was just like reading.

How would you fix them?

I’ll probably put more information on like quitting.

Do you need quitting? How would you change them so they would satisfy you?

Probably like just stay away from them, I don’t know.

Let’s say I wanna fix a video. So you told me there’s too much text. How would you fix that?

I would probably add more games to it.

You like games? What do you like about games?

They like get you to do something, not like just watch the videos, but participate.

Yeah that’s true. If you had like all the money in the world, and you were given the job to fix the website, make it better so that it makes teens not want to smoke, how would you change it?

I will change it to, like, more creative like, I don’t know.

Can you think of something that’s more creative that you’ve seen before? It might help you think of how you wanna make this one?

Not really. I thought the website was pretty good.

What about like if you wanna change some activities. You told me games. Do you have an idea for a game?

Like “What’s the wrong in the picture” games and “cause and effect” games.

Can you tell me more about that?

Like if you did this, then it would end up resulting in like a negative thing. And if did something to like prevent it, it will result in a like more positive thing.

How do you see that helping?

It will help people realize that it’s not a good thing.

Did you feel that ASPIRE made you wanna do something good u haven’t done before?

Yeah, it made me realize that smoking is not something that I wanna do. It’s like something I wanna avoid.

And did you fell that you wanted to do something about it?

Like avoid it.

**15**

**Male**

What did you think of ASPIRE? What was your first reaction on it?

It was interesting but it was just pretty long. That’s it.

What was interesting about it?

Just the animations and everything, and the mini games and everything.

Did you feel like there were games, more than games, less than games, more like activities?

More like activities yeah.

What gives you that hint instead of games?

Because like games make you do certain thing that gives you information on it too, helps you in certain things like that.

So it helps you with information. What was favorite activity?

I don’t know. They all are pretty good actually. They all felt like the same thing.

Like did you have a favorite?

They weren’t all bad. They were all pretty good.

Okay but if you had to pick like the best in your mind, you favorite, your personal favorite?

Probably the best was the one when you click on the cigarette and it goes more down and down and it shows how bad, the hazards in the cigarettes, the screen and what’s inside the cigarette.

What was your least favorite activity?

Find the little film things. You know like where you had to find the cigarette in the movie casting and all that. That was difficult that’s all. It was hard to find.

Why is that?

I had a hard time trying to find it. And it bothered me.

What is your most favorite video?

With the comedian lady, Rena. Pretty funny and just like how she was saying that it could happen to anybody even though she never smoked in her life and everything.

What was your least favorite video?

The one when the girl came home and she had to like call her friends and say that all that other stuff. She said that, well she should have calmed down and then, it was not about what she was going to say.

Why do you like about it?

It kinda didn’t make any sense. It was king of boring.

If u had all the money in the world, and you were invited with a bunch of friends to make ASPIRE program better, what would you add to it?

Like games, more entertaining, instead of going through all these videos. Having little activities, like little games.

What’s special about games?

It just keeps the mind entertained instead of u having like to drag the videos. You keep focus and you have things to look forward to a little bit instead of having the quizzes to look forward to.

Adjective of ASPIRE? Funny, interesting, lot of interesting facts I didn’t know.

**16**

**Male**

What did you think of ASPIRE program?

It’s pretty good for young kids to encourage them not to smoke.

What’s good about it?

It shows you the effects of smoking can do to you.

Like what?

Lung cancer, lip cancer, and stuff like that.

What was your least favorite video?

When the little brother was smoking and the older brother was trying to tell him to stop. And he wouldn’t listen.

Why was it your least favorite?

Because the little brother is always supposed to listen to the big brother.

Ah ok so it wasn’t really your least favorite but it was kinda the one that annoyed you. But I was asking like about the one that was the most boring, the one that you wouldn’t recommend us having?

I would say probably the one with the blue dude. I think he was sitting on a coach watching tv. It was so boring.

Was it about what he was watching?

I don’t remember but I mean I didn’t like the video.

What did it have?

I don’t know.

What was your most favorite video?

The one with the kid who’s seating on the baseball stands.

What was your least favorite boring activity?

The one where you had to click. The pills that you had to click. Red and yellow.

What was bad about it?

I just didn’t get it.

What was your favorite activity?

The one with the clicking of flushing down.

What also did you like?

It’s the activity where you had to click and I liked the one about sport and it showed the difference between if you smoke and if you do not smoke.

If you had all the money in the world, and you were given, you and your friends the opportunity to play around with ASPIRE, make it different, change it, add something to it or take out something to it, what would you do to it? If you had to add something, what would you add?

Probably like more catchy games. Adventure games with rewards.

I would start with the actress, probably like comedian, sports player so people talking.

I just remembered that I liked another video. When that lady comedian was talking about the way she caught it without even smoking.

**17**

**Female**

What did you think of ASPIRE?

It was pretty good. It makes me want to talk with my brother the first time I finished it. Because he smokes so I want to go tell him about some of the facts.

And what happened?

I see that he’s trying to slow down a little bit now.

That’s great! I what did you like most about ASPIRE?

The videos

Which one was your favorite video?

The one with the pink hair on the phone who had trouble with her friend.

What’s special about that video?

When she first called her friend she didn’t think about what she was doing to say. And then again, she calmed herself down and thought about what to say to her friend.

What did it teach you?

It taught me to think before you speak.

What was your least favorite video?

I don’t know. I liked all of them.

But if you have to pick a video that you want us to take out, what would it be?

I forgot it, all I know is there was a boy who was playing basketball or something. That’s all I remember.

What didn’t you like about it?

That it didn’t describe like much.

If you had to pick your favorite activity in there? What was your favorite activity?

The one where you had to pick like the little pictures and it tells you what the warnings are and all.

Which one?

The one where you had to pick like, there’s the cigarette, cigarillo, ..

Oh the different types of smoke and it tells you about each?

Yeah

That was your favorite? What was special about it?

It told you like what they are made of, what’s the warning of it.

And if you had to pick your least favorite activity?

Where you choose the effect of smoking, with different color next to each other, and like when you click on them, they turn yellow.

Do you have another favorite that you liked?

The one where he has to take a puff of the cigarette and he tells you what’s inside of it.

What’s special about it?

That it shows you what’s inside the cigarette and what it’s made of. It shows like the chemical that could cause death or cancer.

If you had all the money in the world and you were with a bunch of friends, and you were asked you and your friends to play around with ASPIRE, change it, add something to it or take something of, what would you add?

I would add like activity where you click each part of your body and it tells you how can the cigarette could affect that piece of your body.

What would you take out?

And I would remove some of the videos to add more activities for the kids to be entertained, What kind of activities?

Like different activities that show the effect, to show why some people think smoking is cool and other can be about what’s really bad about it.

So you want to turn some of the videos to activities?

Yeah.

Because I remember some videos have that, that show why teens think smoking is cool and another show what’s bad about it. But you want to turn them to activities instead of being videos.

What else would you like to tell me about it?

If you get more people to do ASPIRE, they might slow down the smoking. Cause it’s a very good thing.

How old are you?

13.

**18**

**Male**

What did you think of the ASPIRE website?

I actually really liked it. It was organized, easy to learn from and pretty cool.

What did you like most about it?

I liked the fact that they tried to make it for kids. They tried to make it relatable. Better way to understand it for kids our age instead of making it more adults, more funny and everything.

So it’s better tailored for your age?

Yeah

What was your favorite video in it?

When that little kid, his big brother told the little kid about how he used to smoke and stuff and how he thought it was cool. He was actually laughing at him. So he just stopped smoking. It became easier.

And what did you like about it?

I liked the fact that it shows that u don’t have to fit in. Smoking isn’t something that is supposed to make you cool. It somthg that actually hurts u so you should stay away from it. That’s how it was at the beginning. U had to do it to fit in. no peer pressure until you do something about your health first.

What was your least favorite video?

I didn’t have a least favorite.

But if you were asked to remove one, which one will it be?

I think it was when they had that band and then the drummer started smoking when he was supposed to be playing.

And what about the activities, which one was your favorite?

When it showed the different types of tabacco you can smoke besides cigarettes. And how cigarillo and hooka (the products) can also hurt you just like cigarettes can hurt you. The risks are the same. That one was my favorite.

What was special about it?

Because it actually showed that no matter what type of drug you’re doing like there’s no alternative for smoking cigarettes. Like no matter what you’re doing, you’re harming yourself.

What was your least favorite activity?

It was the blue man standing with this long list of things that you get from cigarettes like you’re craving cigarettes, you know what i’m talking about?

What are the colors of the activity?

Its blue and it has like a blue standing over there.

What did you not like about it?

It’s a very long list! I mean it was worth it but it’s too long of a list.

If you wanted to think more about what you liked about it? What’s special about it?

I liked that they showed everythg, the side effects, the symptoms. Craving cigarettes, dizziness, bad breath. Mm all these symptoms.

If you and a bunch of friends had all the money in the world and you were given a chance to change things in ASPIRE program, what would you change? What would you add first? Would you present things in a different way maybe?

The cartoons make it relatable for us. But still, it can go to real life and it can bring people like it has things with real life people. I won’t add or take away anything from it.

What about real people?

Like adding the cartoons, we can relate. Then you have people from the real world, you can actually share their story and how hard it was for them to quit, how they quit and how they celebrate quitting and how they got to do the slipping and all that.

How do you feel ASPIRE might have changed something in your life?

It showed me that no matter what, no matter who offers you something, you do not smoke. Don’t smoke.

What would you do now that you have seen ASPIRE? Something you haven’t done before?

I would actually talk to somebody about smoking and why they shouldn’t do it. I don’t like cigarettes, I’ve got asthma so I get around cigarettes and smell cigarettes, it gives me a headache. But usually I would move around and I wouldn’t say anything. If I know the person, I feel like they’re harming themselves and I care about their well-being, I would actually talk to them now.

And what’s your age?

16.

**19**

**Female**

What did you think of ASPIRE?

It was cool. It was fun.

It was fun? What was cool and fun about it?

They showed you about how people get when they’re smoking and they teach you like things that you shouldn’t smoke and it’s bad for you and they show you like videos, stuff like how to get your body built up and it will make you wanna stop, or it make you don’t, you know, don’t wanna smoke, and I mean it was cool like the videos were cool like the boy and I guess his brother was telling him about smoking and stuff. It was cool. I liked it.

So what was your most favorite video in it?

The video with the blue boy I think yeah that one.

Which one of them?

I think the one where he was in the restroom. It was funny.

And what was your least favorite video?

The one where it showed how your body will look, and how it had to do surgery on people and stuff like that.

Why was it your least favorite?

Cause I don’t like looking at stuff like that. It was kinda not good to look at.

But what about the video that you feel like we’re supposed to take out/delete/remove from ASPIRE?

There wasn’t really none. They were all pretty good.

Would you want us to take out the one with the graphics?

Yeah

You don’t think that it’s gonna help people look at things differently?

I mean, it might but not for me.

Okay and what was your most favorite activity?

The one with the, it was the one with the cards, it was last week, the cards, it was the man with blond hair. It was telling us something about smoking and stuff. It was like a game that we had to play.

So what would you do in the game?

We had to like answer the questions. It referred back to the video with the blue guy.

Is it the video where they offer you a cigarette?

Yeah I think it was that one. They offer you a cigarette.

Is that it? The guy was blond?

Yeah I think so.

I don’t remember anyone. Okay what was your least favorite video?

I don’t really have a least favorite video. I liked all of them except the graphic one. I liked all of them.

And what would ASPIRE make you wanna do that you wouldn’t have done before?

Like try not to let people influence me to do things, and like stay with the positive. Be positive basically and, you know, just wanna do right not, cause smoking would like, would do a lot of damage to your body and it will kill you and I would like to live a long healthy life.

Alright. Here’s the last question. If u and like three of your friends were given all the money in the world to make ASPIRE better, what would you do to it?

I guess write more videos for people to look at and make it funner.

How?

Like make the video where the kids can actually do it. And I guess I could write more things to it. Like the games and activities. They can actually play them, not on the computer but like play them.

Give me an example.

Like boarding card games, stuff like that.

In real life?

Yeah, basically, yeah.

**20**

**Male**

What did you think of ASPIRE, the website?

It was good. It taught us not to smoke.

I know that! What was good about it?

It encourages teens no to smoke.

Did you like something specific in it?

How it was telling how to get harmy about it.

What was your most favorite video in it?

I don’t remember. There was a lot of them.

So what’s the first one that comes to your mind and you like it?

I didn’t like it but it was the first one I remember. The one with people and parts of their body and their face.

Oh the people with like cancer and stuff? The graphics? Ok. If you want to think of a second one that comes to your mind, what would it be?

The one about the little boy and his big brother trying to teach him about the bad things about smoking.

Yeah that’s one of my favorites. And what was your most favorite activity?

The one with the lady and the genie ball.

What was your least favorite activity?

The one where you had to click on all the things. It tells you all the moods.

How does it look like?

Like, oh no that’s not the one. It was the one where it was the people where you could go to for help. You could talk to when u have a problem.

People you can go to for help? Ok yeah. I see how it’s annoying. What was bad about it though?

It took forever.
